# Fuzzy State Transition and Kalman Filter Applied in Short-Term Traffic Flow Forecasting

**DOI:** 10.1155/2015/875243

**Published:** 2015-12-08

**Authors:** Deng Ming-jun, Qu Shi-ru

**Affiliations:** ^1^School of Automatic Control, Northwestern Polytechnical University, Xian 710072, China; ^2^School of Civil Architecture, East China Jiaotong University, Nanchang 330013, China

## Abstract

Traffic flow is widely recognized as an important parameter for road traffic state forecasting. Fuzzy state transform and Kalman filter (KF) have been applied in this field separately. But the studies show that the former method has good performance on the trend forecasting of traffic state variation but always involves several numerical errors. The latter model is good at numerical forecasting but is deficient in the expression of time hysteretically. This paper proposed an approach that combining fuzzy state transform and KF forecasting model. In considering the advantage of the two models, a weight combination model is proposed. The minimum of the sum forecasting error squared is regarded as a goal in optimizing the combined weight dynamically. Real detection data are used to test the efficiency. Results indicate that the method has a good performance in terms of short-term traffic forecasting.

## 1. Introduction

As China's economy improves and urbanization increases, traffic problems intensify while the transportation system develops rapidly. Traffic problems emerge because of a drawback in the development of urbanareas. Developing urban intelligent transportation systems (ITS), which are an effective method for alleviating urban traffic problems, is necessary. However, a basic requisite of ITS is short-term traffic flow state. The information on futureshort-term traffic state can be used in real-time traffic control, real-time traffic induction, and other related processes.

Fortunately, traffic flow exhibits strong randomness, good regularity, and statistical properties. Therefore, in taking advantage of these properties, using the historical traffic state data allows the estimation of the short-term traffic state. Traffic flow is an important parameter for traffic state; thus, this study describes the forecasting method of short-term traffic flow.

Recently, many scholars have studied this issue, and a significant number of methods exist in the field. These methods include spectral analysis method [1], time series model [2, 3], KF methods [4], nonparameter regression methods [5], neural network methods [6], chaotic theory [7], fuzzy logic system [8], and wavelet transform models [9].

Although exhibiting superior capability for forecasting, all of the abovementioned review methods still have several flaws for complex and volatile traffic states. That is, these models have their own advantages, disadvantages, and suitable conditions. For example, spectral analysis methods need the decomposition of traffic state series, which would increase the difficulty of many calculations. Time series models constantly demonstrate good performance for stable traffic conditions but cannot reflect the dynamic, stochastic, and nonlinear properties of traffic flow. KF exhibits high prediction precision, but it is a linear forecasting model and is unfit for nonlinear traffic flow; hence, the method cannot always adapt to variable traffic conditions. Furthermore, sometimes KF prediction values may be delayed compared with the real values. Nonparametric regression method is suitable for short-term traffic dynamic and nonlinear features, but it requires a large amount of historical data. Moreover, many computational resources are required. Neural networks and other intelligent learning algorithms are not based on a theoretical model. By training from a part of historical data, these neural networks and other intelligent learning algorithms can dictate the relationship between inputs and outputs. This advantage of predicting traffic states can exhibit good accuracy in general. However, some example tests show that training takes time; moreover, the results in some contexts easily fall into local minimal, aside from poor generalization. Chaos theory can present the nonlinear features of traffic, but the model parameters such as delay time and embedding dimension are difficult to determine. Only the use of fuzzy state on forecasting can estimate traffic state trends, but its accuracy is not very good. Wavelet transform forecasting efficiency is influenced by the decomposition and reconstruction of series, thus engendering fluctuation in the performance.

To improve the strong adaptation of traffic forecasting models, several integrated models have been further developed. These new models are chiefly based on integration and combined thinking. Some instances combine wavelets with nonparameter regression or Auto Regression Move Average model (ARMA) [10, 11]. By contrast, other instances combine neural networks with fuzzy logic, and so on. Combination models combine a variety of forecasting submodels integrated together for different traffic conditions are composed of those parts that perfectly fit the submodel to predict the current traffic state, thus effectively increasing the adaptability of the model and improving the accuracy [12]. Combination modes are generally presented as the weighted summation of submodels. One submodel may be more accurate at one time or a traffic condition may not be so perfect at another time. Therefore, the weight dynamically adapts according to the traffic state, which may optimize the efficiency of the combined model. Wang et al. employed Bayesian theories to modify the combination weights using the previous prediction precision model. The model is composed of the Back Propagation (BP) neural network model and the Auto Regressive Integrated Moving Average (ARIMA) model. According to the performance of the practical traffic data prediction, the results of the combined model are better than those of the single prediction model [13]. Nonparametric regression and moving smoothing method have been combined by Ying-hong et al. to be applied in short-term traffic prediction. This example demonstrates that the average absolute relative deviations of the methods are all less than 10% [14]. Xiangjie proposed a fuzzy intelligence combined method that includes the KF model, Artificial Neural Network (ANN) model, and fuzzy logic combination model. Practical application results indicate that the combined model, which takes advantage of the unique strength of the KF model and the ANN model, can produce more precise forecasting than that of the two individual submodels [15].

Although traffic flow may be affected by weather and traffic incidents, in general, traffic flow in an average road exhibits strong, long-term statistical characteristics. Therefore, based on statistical thinking, combining the state transition probability and KF theory to predict short-term traffic flow is feasible. This study is based on the two theories to introduce the realization method and its efficiency.

## 2. Fuzzy State Transition Prediction Method

Two contents are introduced in this section. One is the fuzzy state transition forecasting idea and means. The other is updating the state transfer matrix and constantly making the matrix agree with the currently varying patterns of traffic.

### 2.1. Fuzzy State Transition Prediction Model

In an average road, traffic flows from one state to another and displays strong statistical properties. Therefore, according to the fluctuation range, the historical traffic flow data will be divided into *K* fuzzy states. Thereafter, with the aid of the member function, the parameter's membership value (*μ*_*k*_, *k* = 1,2,…, *K*) in state *k* can be determined. The maximum membership degree corresponding to the state is defined as the state values of the current parameters. The current detection flow data combined with the historical transferring laws among those *k* states can construct a *K∗K* transfer matrix *p*_*st*_ as seen in

(1) pst=p11p12…p1Kp21p22…p2K…………pK1pK2…pKK,s=1,2,…,K,  t=1,2,…,K,

where *p*_*st*_ = *c*_*st*_/*c*_*s*_. *c*_*s*_ is the number of *s* states appearing in the historical state series. *c*_*st*_ is the number of times that the *s* state appeared in the current state and transformed to the *t* state in next step. The defined membership vector of each state at time *i* is *F*(*v*_*i*_) = {*μ*_1_(*v*_*i*_), *μ*_2_(*v*_*i*_),…, *μ*_*K*−1_(*v*_*i*_), *μ*_*K*_(*v*_*i*_)}. According to the state transfer principle, the step of the state membership degree vector at time *i* + 1 can be presented as *F*(*v*_*i*+1_) = *F*(*v*_*i*_)*∗p*_*st*_. Therefore, the maximum membership degree method is used, or the state of step *i* + 1 can be obtained; thereafter, the corresponding flow volume parameter vector can be obtained at step *i* + 1 forecasting flow *v*_*i*+1_. See the following:

(2) Fvi+1=Fvi∗pst,vi+1=VFvi+1T∑k=1Kμkvi+1,

where *V* denotes the flow parameter vector corresponding to each state maximum membership degree.

### 2.2. Updating the Status Transfer Matrix

The status of road traffic changes over time; thus, the transfer matrix needs to be updated in real time. Actually, a transfer event has the least influence on the transfer matrix. Therefore, the transfer matrix update rules should be determined to reduce unnecessary calculations. Moreover, the transfer matrix can keep up with the traffic state transformation properties. For *h* consecutive times, step prediction errors are viewed as a signal for a matrix update. Threshold *ε* is defined as follows. If the error is greater than *ε*, then the matrix would be updated; otherwise, the current matrix is retained. Excessive historical data on detection would influence the current prediction accuracy; therefore, some data that are too old should be omitted. The series of historical data brings the length parameter *θ*. When the transfer matrix is updating, *θ* time steps of historical data can be used as base data to construct the new transfer matrix. The values of *h*, *ε*, and *θ* can be decided by road traffic properties separately.

## 3. Kalman Filter Model

In considering that traffic has similar characteristics for one link on the same day in different weeks, the day at times *t*, *t* − 1, *t* − 2,…, *t* − *N* + 1 is set; the historical flows of the road are *V* ^*b*^(*t*), *V* ^*b*^(*t* − 1), *V* ^*b*^(*t* − 2),…, *V* ^*b*^(*t* − *N* + 1); and the same workday or weekend is searched *N* consecutive flow data series in the historical detection data. Thereafter, the first *H* sets of historical flow sequence with the minimum Euclidean distance between the current sequence are determined. The corresponding averages of *H* sets are presented as $\overset{-}{V}(t)$, $\overset{-}{V}(t-1)$, $\overset{-}{V}(t-2),\ldots ,\overset{-}{V}(t-N+1)$. The equation is as follows:

(3) $$\begin{array}{c} \overset{-}{V}\left(\;t\;\right)=\frac{1}{H}\overset{H}{\underset{h=1}{\sum }}V_{h}^{b}\left(\;t\;\right), \end{array}$$

where *h* is the label of the selection data set that has the minimum Euclidean distance between the current sequences, and *b* is the day, such as Monday, Tuesday,…, Sunday.

The ratio of the current sequence and the mean sequences are presented as *r*(*t*), *r*(*t* − 1), *r*(*t* − 2),…, *r*(*t* − *N* + 1) and are calculated by

(4) $$\begin{array}{c} r\left(\;t\;\right)=\frac{V_{t}}{{\overset{-}{V}}_{t}}, \end{array}$$

where *V*(*t*) is the current day flow at time *t*.

When the value of *r*(*t* + 1) is obtained through the Kalman filtering method, *V*(*t* + 1) of the current day can be predicted according to the equation $V(t+1)=\overset{-}{V}(t+1)*r(t+1)$.

*r*(*t* + 1) is assumed to be composed of the previous time steps ratios and is defined as

(5) rt+1=rtxt+rt−1xt−1+rt−2xt−2+⋯+rt−N+1xt−N+1+ωt,

where *ω*(*t*) is the residual. For convenience of expression, (6) are set:

(6) At=rt,rt−1,rt−2,…,rt−N+1,Xt=xt,xt−1,xt−2,xt−N+1T.

Moreover, (7) is set:

(7) $$\begin{array}{c} X\left(\;t\;\right)=B\left(\;t\;\right)X\left(\;t-1\;\right)+w\left(\;t-1\;\right), \end{array}$$

where *B*(*t*) is a state transition vector with a value of 1. *w*(*t* − 1) is the model noise, which is assumed to be zero. For the mean white noise, its covariance matrix is presented as *Q*(*t* − 1).

If *r*(*t* + 1) is set as observation variable *y*(*t*), then the KF state equation and observation equation can be obtained, as seen in the following:

(8) Xt=BtXt−1+ut−1,

(9) yt=AtXt+ωt,

(10) Xt ∣ t=Xt ∣ t−1+Kt·yt−AtXt ∣ t−1,

(11) Xt ∣ t−1=BtXt−1,

(12) Kt=pt ∣ t−1ATt·Atpt ∣ t−1ATt+Rt−1,

(13) pt ∣ t−1=Bt−1pt−1BTt−1+Qt−1,

(14) pt=I−KtAtpt ∣ t−1,

(15) p0 ∣ 0=p0,

where *K*(*t*) is the Kalman gain; *p*(*t*) is the filtering error variance; and *R*(*t*), *Q*(*t*), and *p*(0) can be set to 1 or unit diagonal matrix if no prior data exist.

From the preceding equation, when *X*(*t*) is obtained, then *r*(*t* + 1) is obtained using (9). Formula can present as

(16) $$\begin{array}{c} r\left(\;t+1\;\right)=A\left(\;t\;\right)X\left(\;t\;\right). \end{array}$$

The prediction flow at *t* + 1 is presented as

(17) $$\begin{array}{c} V\left(\;t+1\;\right)=A\left(\;t\;\right)X\left(\;t\;\right)\overset{-}{V}\left(\;t+1\;\right). \end{array}$$

## 4. Combination Forecasting

The state transition model performs well in the prediction of sequence fluctuation trends. However, the essence of the model is the mean probability to base data statistics. Therefore, the prediction result value is always located at the mean of the state. The forecasting accuracy does not completely satisfy the traffic control or guidance requirements. The KF has good performance on linear system estimations. A linear system estimation problem usually obtains accurate results. The KF model is used to forecast the time series problem; the essential forecasting value of *t* + 1 is an extension of time *t* or before the time changes state. Therefore, the KF model is used to forecast nonlinear traffic flow. The result always shows a trace of time *t* or before. The test shows that only when the KF model is used does the forecasting accuracy fail to meet the requirements. However, the state transfer matrix model is used in adjusting the KF. Linear properties may reduce the drawbacks of the two models and improve prediction performance.

The prediction results of each *J* method are assumed to be *y*_*t*+1_^*j*^, *j* = 1,2,…, *J*, and then the combination integration model can be expressed as (18). *y*_*t*+1_^*∗*^ is the forecasting flow at time *t* + 1.

(18) $$\begin{array}{c} y_{t+1}^{*}=\overset{J}{\underset{j=1}{\sum }}\alpha_{t+1}^{j}*y_{t+1}^{j}, \end{array}$$

where *α*_*t*+1_^*j*^ is the weight of each subprediction model at time *t* + 1, which is adjusted by *M* time steps prediction errors before the current time *t*. The submodel that has more errors before the time steps will have a lower weight in the next time step. Based on the preceding concepts, a new combined weight optimization method is developed. The sum of the square of the previous *M* steps' minimum integration errors is obtained as the optimization object, and the optimization submodel weight is subsequently solved. The equation is expressed as follows:

(19) min⁡z=∑i=t−M+1tyi−∑j=1Jαij∗yij2,

(20) ∑jJαij=1,

(21) αij∈0,1,

where *α*_*i*_^*j*^ and *y*_*i*_^*j*^ are defined as the abovementioned contexts, *y*_*i*_ is the detection flow at time *i*, and *M* is the number of detection flow series before the current time. The model output is the optimization submodel weights from *t* − *M* + 1 to *t*. However, (18) requires the submodel weight of time *t* + 1; that is, it requires the value of *α*_*t*+1_^*j*^. In considering the continuity of traffic condition in the short term, a forecasting model has good accuracy at the current period such that in the next adjacent period, it also has good prediction accuracy. Therefore, previous *M* time steps *j* submodel optimization mean weights are used as the *j* submodel weight at time *t* + 1. Thus, the integration weight at time *t* + 1 is presented as

(22) $$\begin{array}{c} \alpha_{t+1}^{j}=\frac{1}{M}\overset{t}{\underset{k=t-M+1}{\sum }}\alpha_{k}^{j}, \end{array}$$

where *M* denotes the actual road traffic state fluctuations.

Equation (19) is a nonlinear constrained optimization problem. Solving this problem involves two methods: one is to eliminate the constraints and then use the quadratic programming method in solving the problem and the other is to utilize the Particle Swarm optimization (PSO) algorithm. The PSO algorithm is an intelligent searching method with good generality. The PSO algorithm is adopted to solve this problem. Equation (19) is considered the fitness function. Constraint conditions (20) and (21) are set as the range of the particles. The particles' speed and location are updated by (23). Meanwhile, the iteration termination condition is set, which is presented as two adjacent iterations of the optimization particles' Euclidean distance that is less than a given threshold *E* or the number of iterations required to reach a certain threshold *G*. When the termination condition is reached, the program exits, and the final output is the optimization of the particles' locations. Particles locations are updated by (24):

(23) vis+1=ωvis+c1ξpis−xis+c2ηpgs−xis,

(24) xis+1=xis+rvis+1,

where *v*_*i*_^*s*^ is the *i* particles' speeds at time *s*; *p*_*i*_^*s*^ is the optimal position of the *i* particles to be searched at time *s*; *p*_*g*_^*s*^ is the globally optimal particles' position at time *s*; *x*_*i*_^*s*^ is the *i* particles' positions at time *s*; *ω* is the inertia weight, which is the weight for the particles to hold their speed; *c*_1_ is the weight of the particles that track their optimal value; *c*_2_ is the weight of the particles that track the global optimal particle position; *r* is the speed constraint factor usually set as 1; and *ξ*, *η* are two uniformly distributed random numbers located at [0,1].

The steps of the algorithm are described as follows.

**Step 1 .**

Randomly generate a certain amount of individual particles that satisfy the constraints condition.

**Step 2 .**

Based on the objective function, calculate the fitness of each particle; update each particle history's optimal fitness value corresponding to the location information; and update the global optimum particle's corresponding location position.

**Step 3 .**

Use (23) and (24) to update the particle speed and position.

**Step 4 .**

Proceed to Step 2 and determine whether to terminate. If the termination condition occurs, then output the final position and proceed to Step 5; otherwise, proceed to Step 3.

**Step 5 .**

Use (22) to calculate *α*_*t*+1_^*j*^.

**Step 6 .**

Use (18) to calculate *y*_*t*+1_^*∗*^, that is, the prediction of traffic flow at time *t* + 1 expressed as *v*_*t*+1_.

## 5. A Practical Case Analysis 

**Figure 1:**
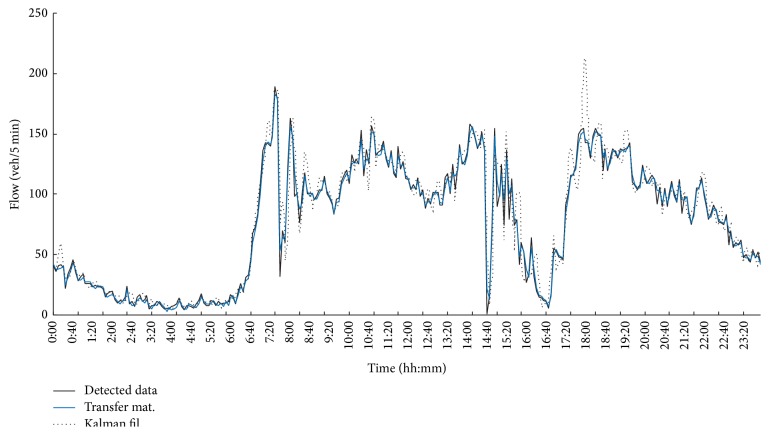
Comparison chart of the two submodels and the real detection data.

**Figure 2:**
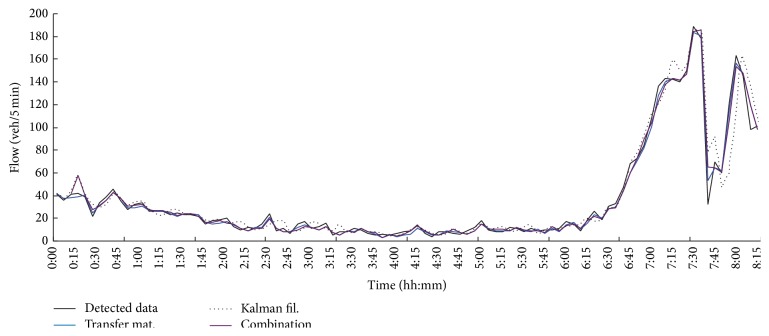
Comparison chart of the combination model and the two submodels.

The continuity flow data series on an expressway in Nanchang shows that the detection interval is five minutes. For four Mondays, these flow data are detected, and one whole day has 288 sequences. All four days include 1,152 flow sequences in total. In this case, the first three Mondays' data are set as experiment data, and the fourth Monday's data are regarded as validation data. First, the transfer matrix method introduced in this study is used to test the efficiency. According to the flow fluctuation range distribution, the flow sequence can be split into 10 fuzzy states *x*_1_, *x*_2_,…, *x*_9_, *x*_10_, and trigonometric functions can be employed as membership functions. Second, the KF method is used to predict the fourth Monday's flow data. Three consecutive time ratios *r*(*t*), *r*(*t* − 1), *r*(*t* − 2) are adopted to construct *A*(*t*). *r*(·) can be calculated by (4). Third, the described combination method is used to predict the fourth Monday's flow data. All three methods have been programmed in MATLAB. The results are presented as flow charts.  Figure 1 shows the fuzzy transfer matrix method prediction flow series curve and the KF. The real detection data are also shown in the figure for comparison.  Figure 1 demonstrates that the two methods have good performance on short-term traffic flow prediction. For further details of each method that include the combination method, clock data for Mondays from 0:00 to 8:00 are used to draw Figure 2. Figure 2 shows that the combination method improved the performance of the two submodels. The combination model's curve is closer to the curve of the real detection data than those of the two submodels taken individually.

In verifying the accuracy of the combination method in this study, the average relative error (ARE), mean square error (MSE), and equal coefficient (CE) are selected as evaluation indicators to examine the performance of the combination method. ARE can predict the extent of deviation of the detected data from the real detection value. MSE reflects not only the deviation extent but also the degree of error of the dispersion. A lower MSE shows a better predicted accuracy. CE reflects the degree of fit of the predicted value and the real detected value. CE should be greater than 0.9; if the value is closer to 1, then it has better prediction performance. In comparing the current method with other short-term traffic prediction methods, the Bayesian combination forecasting model [13] is employed using the same data to predict the fourth Monday's traffic data. The prediction accuracies of the described combination method and the Bayesian combination method are affected by the roll back steps value *M*; thus, different *M* values are set for the two combination models to solve the prediction problem. The performances are shown in the following two tables.  Table 1 presents the two submodels' error indicators, namely, fuzzy transfer matrix and KF.  Table 2 shows the two combination model error indexes under different *M* values.

Each error indicator calculation equation is shown as

(25) ARE=1n∑i=1nv^i−vivi,MSE=1n∑i=1nv^i−vivi2,CE=1−∑i=1nv^i−vi2∑i=1nv^2i+∑i=1nv2i,

where $\hat{v}(i)$ is prediction value at time *i* and *v*(*i*) is real detected value at time *i*.

As the validation data in Tables 1 and 2 show, the combination methods described (PSO) in this paper fit the backtracking time step. Each error indicator is better than the single submodel. For example, the ARE of the transfer matrix model is 9.13%, whereas that of the KF model is 12.43%. When the backtracking time step is equal to 2 (*M* = 2) the combined model performs well; the ARE value is 7.85%. In comparing PSO with the transfer matrix model, 1.28% is optimized, and for the KF model, 4.58% is optimized. The same result is obtained for the Bayesian model combination method. In the Bayesian model, when *M* is equal to 2, ARE is 9.91%, which is the lowest among the AREs. MSE in Table 2 shows that the PSO combination model's values are lower than those of the Bayesian combination model, indicating that the PSO combination method's error distribution is more centralized than that of the Bayesian combination method. Therefore, the prediction reliability of the PSO method is better than that of the Bayesian method. Furthermore, aside from PSO, the Bayesian method generates the lowest MSE when *M* is equal to 2. The CEs of the two methods are above 0.9, and the two methods have the best performance when *M* is equal to 2, similar to ARE and MSE. In terms of integration, the data in Table 2 show that the PSO method's accuracy is better than that of the Bayesian method, and the 2 time steps backtracking is the best fit for the combination model, that is, a 10-minute period is the right evaluation time step for the fluctuation of road traffic flow. The short period then exhibits a specific characteristic; that is, the traffic significantly fluctuates for evaluation. For the long period, traffic stability increases, thus inducing difficulty in predicting traffic.

## 6. Conclusions

The state transfer matrix method used for the time series fluctuation's trend prediction has a good effect. The KF has a good fluctuation in the time series estimation. In this paper, the characteristics of these two submodels are used, and the two submodels are combined. In improving the accuracy of the proposed dynamic combination weight optimization method, which predicts the error square minimum as the optimization objective, PSO is employed to solve the problem. The test results indicate that the two submodels in this study have a good performance in terms of prediction accuracy, but after they are combined, the prediction accuracy is further improved. Compared with the Bayesian combination method, the described PSO combination method performs better.

In conclusion, the numerical analysis confirms that the described method can be applied in short-term traffic flow forecasting. However, these test results also imply that the forecasting does not always have good accuracy, such as when the traffic flow largely fluctuates. Therefore, in the near future, we plan to study the other combination weight optimization methods that can further fully present each submodel's advantage and increase the combination model's universal adaptability.

## Figures and Tables

**Table 1:** Errors of the two submodels.

Method		ARE (%)	MSE (%)	CE
Sub	Trans. matrix	9.13	12.54	0.975
sub	Kalman filter	12.43	22.15	0.959

**Table 2:** Errors of the combination model.

	*M*	ARE (%)		MSE (%)		CE	
		Bay.	PSO	Bay.	PSO	Bay.	PSO
Com.	1	10.25	11.05	19.87	21.42	0.9423	0.9536
	2	9.91	7.85	18.49	16.23	0.9566	0.9673
	3	9.98	8.96	19.65	17.64	0.9531	0.9624
	4	10.22	9.24	19.30	18.35	0.9453	0.9621
	5	10.27	8.95	19.89	17.33	0.9441	0.9623
	6	10.05	8.98	19.08	17.94	0.9451	0.9612
	7	10.07	9.02	19.84	17.77	0.9453	0.9621
	8	10.22	8.87	19.23	17.56	0.9435	0.9625

## References

[1] Nicholson H., Swann C. D. (1974). The prediction of traffic flow volumes based on spectral analysis. *Transportation Research*.

[2] Kamarianakis Y., Gao H. O., Prastacos P. (2010). Characterizing regimes in daily cycles of urban traffic using smooth-transition regressions. *Transportation Research Part C: Emerging Technologies*.

[3] Min W., Wynter L. (2011). Real-time road traffic prediction with spatio-temporal correlations. *Transportation Research Part C: Emerging Technologies*.

[4] Okutani I., Stephanedes Y. J. (1984). Dynamic prediction of traffic volume through Kalman filtering theory. *Transportation Research Part B*.

[5] Jia N., Shou-Feng M. A., Zhong S. Q. (2012). Non-parameter-regression traffic flow forecast method based on KD-tree and genetic optimization. *Control and Decision*.

[6] Vlahogianni E. I., Karlaftis M. G., Golias J. C. (2005). Optimized and meta-optimized neural networks for short-term traffic flow prediction: a genetic approach. *Transportation Research Part C: Emerging Technologies*.

[7] Hu J., Zong C., Song J., Zhang Z., Ren J. An applicable short-term traffic flow forecasting method based on chaotic theory.

[8] Zhang X., Onieva E., Perallos A., Osaba E., Lee V. C. S. (2014). Hierarchical fuzzy rule-based system optimized with genetic algorithms for short term traffic congestion prediction. *Transportation Research Part C: Emerging Technologies*.

[9] Xie Y., Zhang Y. (2006). A wavelet network model for short-term traffic volume forecasting. *Journal of Intelligent Transportation Systems: Technology, Planning, and Operations*.

[10] Gao Y., Chen F. (2008). Wavelet analysis-based npr prediction of short-term traffic flow. *Journal of University of Science & Technology of China*.

[11] Dou H., Liu H., Wu Z., Yang X. (2009). Study of traffic flow prediction based on wavelet analysis and autoregressive integrated moving average model. *Journal of Tongji University*.

[12] Nie P. L., Zhi Y. U., Zhao-Cheng H. E. (2008). Constrained kalman filter combined predictor for short-term traffic flow. *Journal of Traffic & Transportation Engineering*.

[13] Wang J., Deng W., Zhao J. (2012). Short-term freeway traffic flow prediction based on improved Bayesian combined model. *Journal of Southeast University*.

[14] Ying-hong L., Le-min L., Yu-quan W. (2013). Short-term traffic flow prediction based on combination of predictive models. *Journal of Transportation Systems Engineering & Information Technology*.

[15] Xiangjie S. G. X. (2011). Short-term traffic volume intelligent hybrid forecasting model and its application. *Systems Engineering—Theory & Practice*.

